# The Sociodemographic Determinants of Nurse Exportation: An Analysis of the Willingness of Ghanaian Nurses for Overseas Employment

**DOI:** 10.1155/jonm/8819130

**Published:** 2026-02-03

**Authors:** Emmanuel Nyameke, John Ansah, Isaac Defiin, Alex Boadi Dankyi, David Doku Teye

**Affiliations:** ^1^ Department of Sociology and Anthropology, University of Cape Coast, Cape Coast, Ghana, ucc.edu.gh; ^2^ Research Fellow at the University of Cape Coast, University of Cape Coast, Cape Coast, Ghana, ucc.edu.gh; ^3^ Department of Population and Health, University of Cape Coast, Cape Coast, Ghana, ucc.edu.gh

**Keywords:** government, nurse export, nurses, sociodemographics, willingness

## Abstract

Nurse exportation, driven by sociodemographic factors, has become a significant trend in many African countries, including Ghana. Factors such as age, gender, marital status, religion, and family responsibilities influence nurses’ willingness to migrate for better economic and professional opportunities. This study explores the sociodemographic dimensions shaping nurse exportation in Ghana, employing a mixed‐methods approach with a sequential explanatory research design. A cross‐sectional survey of 483 nursing students and interviews with seven nurses provided insight into migration patterns. The findings indicate that younger nurses (18–25 years) exhibit a higher willingness to migrate, driven by aspirations for career advancement and financial stability. Gender disparities also play a role, with male nurses facing fewer mobility constraints compared to their female counterparts. In addition, marital status and religion were not found to significantly influence migration decisions, while having dependents emerged as a critical factor, with nurses without children being more likely to migrate. Given these findings, this study recommended that stakeholders prioritize younger nurses for international opportunities, as they exhibit greater flexibility and willingness to relocate. In addition, policies should be developed to support nurses with dependents, such as structured relocation assistance and family reunification programs, to facilitate their participation in overseas employment opportunities.

## 1. Introduction

Nurse exportation, that is, the migration of nurses from one country to another, is often driven by various factors, including sociodemographic influences. Sociodemographic factors encompass population characteristics such as age, gender, education, employment status, and social conditions, which significantly shape migration patterns in the healthcare sector, including the migration of nurses​ [1]. These factors significantly affect nurse exportation, particularly in countries experiencing workforce imbalances. Again, the expectation for better income in the destination countries has been regarded as one of the motivating factors of migration among nurses.

In Africa, particularly in countries such as Ghana, Nigeria, Malawi, and Kenya, among others, workforce imbalances within the health sector have been on the rise due to various factors, including population growth, economic constraints, and inadequate health infrastructure. As a result, nurse exportation has emerged as a viable strategy to address these imbalances by providing trained nurses with opportunities abroad while also alleviating workforce saturation in their home countries. This phenomenon has been particularly evident in nations where the supply of trained nurses has exceeded local demand or where financial constraints have limited the ability of governments to absorb newly trained healthcare professionals into the system.

For example, in Kenya, the number of trained nurses has steadily increased over the years due to improved educational opportunities and government initiatives aimed at expanding the healthcare workforce. However, the local health sector has faced challenges in employing all these professionals, leading to a surplus of qualified nurses. To address this, the exportation of Kenyan nurses to countries such as the United States of America, the United Kingdom, Canada, and other European nations has been actively pursued as a means of balancing workforce supply and demand [2]. These destination countries often experience nursing shortages due to aging populations, increasing healthcare needs, and declining numbers of locally trained professionals [3]. Consequently, Kenyan nurses, along with their counterparts from other African countries, have been recruited to fill these gaps, benefiting both the host and source countries.

While nurse exportation has been widely regarded as a practical solution to workforce imbalances and a means of providing better economic opportunities for health professionals, sociodemographic factors have also played a crucial role in shaping this trend. One significant demographic characteristic that influences the dynamics of nurse exportation is gender differences. Nursing, traditionally perceived as a female‐dominated profession, often sees women making up the majority of the workforce. However, cultural norms, family responsibilities, and migration policies can impact the willingness and ability of female nurses to seek opportunities abroad [4]. In some cases, male nurses face fewer societal and familial restrictions when migrating for work, which can lead to variations in the composition of exported nursing professionals. In addition, the demand for nurses in destination countries may be influenced by gender‐based preferences, with some healthcare systems favoring male nurses for specific roles, particularly in settings where gender dynamics play a role in patient care.

In Ghana, the government has faced challenges in integrating all trained nurses into the local healthcare system due to limited employment opportunities, financial constraints, and structural inefficiencies. The inability of the Ghanaian government to absorb all trained nurses into the local healthcare sector has led to an increasing surplus of qualified but unemployed or underemployed nursing professionals. In an attempt to address this challenge, the government has encouraged nurse exportation as a means of providing alternative employment opportunities. However, while this strategy offers a solution to unemployment in the country, sociodemographic factors significantly influencing nurse exportation cannot be overlooked. Gender disparities affect migration decisions, with female nurses often facing greater sociocultural and family‐related constraints when seeking employment abroad, whereas male nurses may experience fewer restrictions. In addition, age, experience level, and education may determine access to international opportunities, creating inequalities in migration patterns. Despite the government′s initiative, little research has been conducted to understand the sociodemographic dimensions of nurse exportation and their implications for the Ghanaian healthcare system. The study therefore focuses on examining the sociodemographic determinants influencing the willingness of Ghanaian nurses to accept to be exported or seek overseas employment. It investigates how factors such as age, gender, marital status, religion, dependents, education, professional background, and income shape a nurse’s willingness to accept to be exported. The primary objective of this research is to determine which sociodemographic variables (for example, age, gender, family dependents, education level, professional specialization, and income) significantly influence nurses′ willingness to accept to be exported.

## 2. Methodology

### 2.1. Research Design

While performing this study, Creswell and Clark [5] served as a guide, directing the methodical process of data collection, analysis, interpretation, and reporting. A mixed‐methods approach and a sequential explanatory design were utilized in this study, integrating both quantitative and qualitative research methods to leverage their respective strengths while mitigating their limitations [6]. This approach was chosen to ensure a comprehensive and well‐rounded analysis of the willingness of nurses to be exported, rather than relying exclusively on either quantitative or qualitative data [7]. By combining quantitative and qualitative approaches in this study, the study presents both the numbers as well as the meaning behind the display in the tables. This methodological choice aligns with previous scholarly recommendations [8–10], which emphasize the effectiveness of mixed‐methods research in capturing rich, multidimensional insights. By integrating both data types, this approach provided a broader and more detailed perspective on the subject under investigation.

After defining the research problem and methodology, researchers adopted a sequential explanatory research design. This approach began with quantitative data analysis to establish a broad understanding of the topic, followed by qualitative analysis to refine and explain the findings, particularly from the perspectives of participants [11]. The sequential design not only validated and enriched the data but also helped identify contradictions and explore divergent viewpoints.

As Cresswell and Clark [12], observed, by the sequential explanatory design, we collected quantitative data to assess whether there are some relationships between the sociodemographic variables of the nurses and their willingness to accept to be exported. So, for example, the data measured the age categories of nurses and their influence on a nurse accepting to be exported. By that design, we further interrogated some younger and older nurses, as per the age distributions, as to why they will or will not accept to be exported.

Following the sequential explanatory design, after we had analyzed the quantitative data, we went ahead to further engage some nurses who had taken part in the quantitative data collection in‐depth, following how the quantitative data showed. This helped to understand the meaning behind some of the statistics, as shown in the chi‐square analysis and the regression.

### 2.2. Study Area, Population, Sample Size, and Sampling Procedure

This study was conducted in Cape Coast, Ghana. Researchers targeted three nursing training institutions. The three nursing training colleges were selected because they were the only three government colleges in Cape Coast where the study was being conducted. A cross‐sectional survey was conducted with 483 respondents from three different nursing training institutions with different concentrations of training, and the data were triangulated with qualitative data using interviews with 7 nurses who responded to the questionnaire. The lottery technique was used to select individual respondents. Thus, the researcher wrote “yes” and “no” on pieces of paper and presented the papers to the students. Those students who picked “yes” were included in the study.

### 2.3. Sample Procedure

Recruiting research respondents and participants is key to the research methodology. For the purpose of the survey, in order to obtain the sample size for data collection, the researcher used the Slovin’s sample size formula [13] as

(1) n=N/1+Nsquare of e,

where “*n*” is the required sample size, “*N*” is the total population, and “*e*” is the acceptable margin of error.

According to the Department for Adult Health (University of Cape Coast [UCC]), the total population of final‐year nursing students at UCC was 136. According to the formula, a sample size of 104 was obtained from the total population of the class. The person in charge of academics provided the total population for Cape Coast Nursing and Midwifery College (CCNMC). The total sample of final‐year students at CCNMC is 372, of which the sampled number for this study was 193. The total population of final‐year students of psychiatric nursing training, Ankaful, was 351, as quoted by the staff in charge of academics, and after computation of the figures into the formula, the sample for the survey was 187. Putting the individual sample size together, the total sample size for the quantitative data was 483.

### 2.4. Data Collection, Analysis, and Presentation

SPSS Version 26 was used to process the data. Analyzing the data, the researcher conducted measures of frequency to describe the sociodemographics of the respondents. Testing the hypothesis, the researcher ran a crosstab analysis and included the chi‐square analysis to check how the sociodemographic characteristics of nursing students influence their willingness to be exported. The quantitative data were triangulated with the qualitative data. After the initial analysis of the hypothesis was performed and the age and number of children of respondents showed some relationship with their willingness to be exported, a multinomial logistic regression analysis was run on the age and number of children and their association with the willingness of nurses to be exported, as well as to establish which variable was able to predict their willingness to be exported. The qualitative data were analyzed using thematic narrative analysis. In this paper, the qualitative data have been integrated with the analysis without always using the usual quotation marks and italics for quotes. This change was performed to present the data and the analysis in a more integrated manner.

### 2.5. Quantitative Data Collection Process

A questionnaire was designed to gather data from two different nursing colleges (CCNMC and Pentecost Nursing and Midwifery Training College [PNTC]) and one university nursing college (UCCSNM), of which all were final‐year students. The questionnaire was used to collect data to satisfy Objective 1 and Objective 2. The questionnaires were designed based on a review of the literature and certain variables the researcher considered important to investigate to enrich the data. The questionnaire had 63 items on it. Respondents were to choose their answers between agree and disagree. The first part of the questionnaire focused on sociodemographic characteristics (age, sex, level of education, religion, marital status, and type of profession). In the second section of the questionnaires, questions were asked based on specific themes. The questionnaire covered some issues on training for export, the selection process, the benefits of nurse exportation, and what the government of Ghana should do if nurses are to be exported.

For the quantitative part of the study, surveys were conducted across three institutions: the CCNMC, the UCC, and the PNTC. At CCNMC, the researcher used a lottery system, students picked “Yes” or “No” from a container, and only those who drew “Yes” participated. Although participants had been given fair chances to be part of the study, for the sake of voluntarism, participants who had initially picked yes and may still not want to be part of the study and participants who picked no but wanted to be part of the study were given the chance to make their final decisions to either be part of or not. Doing so made it possible for a few participants who picked yes and did not want to participate to be excluded, and a few others who had picked no but wanted to participate in the study to be included. A total of 193 questionnaires were completed, including responses from general nursing students, midwifery students, and workers on study leave. Similarly, at UCC, final‐year nursing students gathered in the library, and 104 respondents were selected using the same lottery method, with all questionnaires successfully retrieved. At PNTC, students who had just finished exams were invited to participate, and 186 surveys were completed after the random selection process. In all cases, tutors assisted in organizing the sessions, ensuring full participation and retrieval of responses.

### 2.6. Interview Process

Before the researcher could conduct the interviews with some of the students, he made them aware that after they had administered the questionnaires, he would need some students to engage them in an interview section after he finished analyzing the quantitative data. So, the researcher found some students who opted to be part of it. However, when the time was due for the interviews as scheduled, the nursing students were very busy with licensure exams, so only a few of them could turn up for the interviews. Overall, seven participants were conveniently interviewed, out of whom three were from the UCC and four (4) were from CCNMC. At CCNMC, the interviews were conducted in the exam room, and at the university, they were conducted under summer hats on the university campus.

## 3. Results

In this study, the willingness of nurses to be exported out of Ghana was analyzed using measures of frequency, chi‐square, and multinomial logistic regression analyses for the quantitative data. The thematic narrative analytical technique was used to present the qualitative data. The thematic presentation of the study findings has been adhered to, focusing on various demographic factors.

### 3.1. Demographics of Respondents

In this study, the researchers deliberately analyzed the sociodemographic factors of respondents. This was performed in a preamble to delve into the respondents’ composition in terms of age, sex, religion, marital status, children, educational level, professional background, income level, and the institutions of the respondents. For instance, age plays a critical role in shaping nurses’ mobility decisions, with younger nurses exhibiting a higher willingness to be exported compared to their older counterparts.

### 3.2. Institutions Composition

Table 1 presents the contribution of the three institutions in this study, based on their sample size. At the UCCSNM, 104 respondents were involved in this study, representing 21.5% of the entire study sample size. Again, from PNTC, 186 (38.5%), and UCCSNM, 193 (40%) of the respondents were involved in this study. The difference in the number of respondents from the three institutions was based on their population.

**Table 1:** Institutions of respondents.

Institutions	Frequency	Percent (%)
UCCSNM	104	21.5
PNTC	186	38.5
CCNMC	193	40

### 3.3. Age of Respondents

As revealed in Table 2, the findings indicate that the majority of respondents (77.8%) fall within the 18–25 age range, classified as youthful by WHO standards, while 22.2% are between 26 and 45 years old. This age distribution has significant implications for nurses′ willingness to be exported. Younger nurses are generally more open to migration, driven by aspirations for career advancement, better remuneration, and international exposure. Their fewer family obligations and greater adaptability make them more likely to seek opportunities abroad. In contrast, older nurses, particularly those within the 26–45 age group, may have established family ties, job stability, or personal commitments, making them less inclined to migrate. A 38‐year‐old participant shared that “I have made my family here in Ghana, and I also have to take care of my aged mother, hence, I cannot leave this huge responsibility in the name of a job abroad.” On the contrary, a 22‐year‐old nurse shared that “I can′t wait to grab the opportunity to work abroad. I have a dream, and I will be glad to be selected to work on a government program.” This finding aligns with the study by Bugri [14] that revealed that young nurses who graduated between 2019 and 2021 are unemployed and have been willing to work outside Ghana in order to support their livelihoods.

**Table 2:** Age of respondents.

Age of respondents	Frequency	Percent (%)
18–25	376	77.8
26–35	95	19.7
36–45	12	2.5

Furthermore, it can be deduced from Table 2 that the youths who fall between the ages of 18 and 25 are energetic people who can migrate. As such, when they are unable to secure jobs in Ghana, they will migrate to other countries. This revelation aligns with a study by Coulibaly and Page [15] and the World Bank report (2021) that unemployed persons between the ages of 15 and 25 have been taking up employment opportunities outside their home countries.

### 3.4. Sex of Respondents

As shown in Table 3, the study revealed that 352 (72.9%) were females, while 131 (27.1%) were males. The gender distribution of respondents, with 72.9% female and 27.1% male, has significant implications for nurses’ willingness to be exported. Given that nursing is a female‐dominated profession, it is expected that more women would consider migration opportunities. However, sociocultural factors, family responsibilities, and gender norms may influence their mobility. Female nurses often face challenges such as spousal and childcare obligations, which can limit their ability to migrate freely compared to their male counterparts. In contrast, male nurses, though fewer in number, may experience fewer societal constraints, making them more likely to seek international employment. Therefore, while female nurses represent the majority, their willingness to be exported may be lower due to personal and societal expectations, whereas male nurses may have greater flexibility in pursuing overseas opportunities.

**Table 3:** Sex of respondents.

Sex of respondents	Frequency	Percent (%)
Females	352	72.9
Males	131	27.1

### 3.5. Religion of Respondents

In this study, the religious affiliation of respondents was computed, as religion has a bearing on the willingness to migrate. Such being the case, respondents through the questionnaire were requested to indicate their religion. While religion may not be a primary determinant, it can play a role in shaping destination preferences and overall willingness to be exported.

As shown in Table 4, this study revealed that 459 (95.0%) were Christians, while 24 (4.8%) were Muslims. The religious composition of respondents, with 95.0% identifying as Christians and 4.8% as Muslims, may have implications for nurses′ willingness to be exported. Religious beliefs and practices can influence migration decisions, as some nurses may prefer destinations that align with their faith and cultural values. For example, Christian nurses, being the overwhelming majority, may find it easier to integrate into Western countries where Christianity is prevalent, making them more open to migration opportunities. Conversely, Muslim nurses, though fewer in number, may consider factors such as religious freedom, availability of mosques, and acceptance of Islamic practices when deciding on migration.

**Table 4:** Religion of respondents.

Religion of respondents	Frequency	Percent (%)
Christian	459	95.0
Muslim	23	4.8
Others	1	0.2

*Note: Source:* Field data, 2023.

### 3.6. Marital Status

As revealed in Table 5, data show that 440 (91.1%) of the respondents were single, while 43 (8.9%) were married. The marital status of respondents, with 91.1% being single and only 8.9% married, has significant implications for nurses’ willingness to be exported. Single nurses are generally more flexible and mobile, as they have fewer family obligations and responsibilities that might hinder migration. Their willingness to relocate is often driven by career advancement, financial opportunities, and personal growth. In contrast, married nurses may face greater constraints, such as spousal commitments, childcare responsibilities, and the challenge of relocating with a family. In addition, married nurses may need to consider their partner’s career and family stability, making them less likely to migrate compared to their single counterparts. Therefore, the high percentage of single nurses suggests a greater overall willingness to be exported, as they have fewer personal barriers to international mobility.

**Table 5:** Marital status of respondents.

Marital status	Frequency	Percent (%)
Single	440	91.1
Married	43	8.9
Total	483	100.0

*Note: Source:* Field data, 2023.

### 3.7. Number of Children or Dependents

As shown in Table 6, data revealed that 434 (89.9%) had no children or dependents, while 49 (10.1%) of the respondents had children or dependents. As the data indicate that 89.9% of respondents had no children or dependents, while 10.1% had familial responsibilities, this has important implications for nurses’ willingness to be exported. Nurses without dependents are generally more open to migration, as they have fewer personal obligations tying them to their home country. They may be more willing to explore international opportunities for career advancement, better salaries, and improved living conditions. On the other hand, nurses with children or dependents may face greater challenges in relocating, as they must consider factors such as childcare, family stability, and support systems in the destination country. The need to balance professional aspirations with family responsibilities may reduce their likelihood of migration. Therefore, the high percentage of nurses without dependents suggests a greater overall willingness to be exported, as they have fewer constraints limiting their mobility.

**Table 6:** Number of children of respondents.

Number of children	Frequency	Percent (%)
None	434	89.9
Children	49	10.1
Total	483	100.0

*Note: Source:* Field data, 2023.

### 3.8. Educational Level

In Table 7, data revealed that 379 (78.5%) of the respondents were pursuing a diploma, while 104 (21.5%) were pursuing a first‐degree level of education, which has significant implications for nurses’ willingness to be exported. Generally, higher educational qualifications enhance job mobility, as degree holders may have greater access to international opportunities, higher earning potential, and more specialized roles in foreign healthcare systems. Diploma holders, while forming the majority, may face limitations in meeting the qualification requirements of certain destination countries, potentially reducing their chances of migration. However, diploma holders may still be motivated to migrate in pursuit of career growth, further education, and better economic prospects. Therefore, while both groups may be willing to migrate, degree holders may have greater opportunities and higher chances of successful nurse exportation due to their advanced qualifications.

**Table 7:** Educational level of respondents.

Educational level	Frequency	Percent (%)
Diploma	379	78.5
Degree	104	21.5
Total	483	100.0

*Note: Source:* Field data, 2023.

The data in Table 8 revealed that 292 (60.5%) were special nurses, 93 (19.3%) were general nurses, 5 (1%) were professional nurses, and 93 (19.3%) were midwives. The professional background of respondents, with 60.5% being special nurses, 19.3% general nurses, 19.3% midwives, and 1% professional nurses, has significant implications for the willingness to be exported. Special nurses, having specialized training in areas such as cardiac or critical care, may have higher international demand, increasing their likelihood of migration for better career opportunities and competitive salaries. General nurses and midwives, though essential, may face greater competition in securing overseas positions, depending on the specific demands of destination countries. The small percentage of professional nurses suggests that those with higher qualifications and broader expertise are limited, which may influence migration trends. Overall, the dominance of special nurses in the sample suggests a higher willingness and opportunity for exportation, as specialized skills are often sought after in international healthcare systems.

**Table 8:** Professional background of respondents.

Professional background	Frequency	Percent (%)
Special nurse	292	60.5
General nurse	93	19.3
Professional nurse	5	1.0
Midwifery	93	19.3
Total	483	100.0

*Note: Source:* Field data, 2023.

Table 9 presents data on the income background of respondents. A total of 412 respondents, representing 85.3%, were not income earners. From Table 9, 71 respondents, representing 14.7%, were income earners. This is the case because these respondents are workers who are on study leave. The majority of the respondents were not income earners. This is because the 412 respondents have never worked, unlike the 71 who were nurses but went to school for a top‐up. For nurses who were working and earning income, 45 of them, making 64.8%, were receiving between Ghc 1000 and 1500 cedis. A few of the nurses making 35.2% were receiving between Ghc 2000 and 3000 cedis. Considering the economic conditions in Ghana and an inflation rate of 25.8% as of March 2024 (Bank of Ghana), nurses will be more willing to accept being exported to destination countries where salaries seem better.

**Table 9:** Income of respondents.

Income	Frequency	Percent (%)
No income	412	85.3
Income	71	14.7
*Salary*		
1000–1500	46	64.8
2000–3000	25	35.2
Total	483	100.0

*Note: Source:* Field data, 2023.

In this study, researchers conducted further analysis of the likelihood of nurses willing to migrate based on sociodemographic factors.

As shown in Table 10, the analysis of sex and willingness to be exported revealed a chi‐square value of 2.642 and a *p* value of 0.104, indicating that there is no statistically significant relationship between gender and willingness to migrate among nurses in Ghana. This suggests that both male and female nurses would be willing to be exported, despite potential societal and cultural influences. While some assumptions may suggest that male nurses face fewer mobility constraints and female nurses may be restricted by family responsibilities, this study revealed that gender alone does not significantly determine a nurse’s decision to migrate. This finding confirms the study by Oishi [16], Kanaiaupuni [17], and Gubhaju and De Jong [18] that migration has not been limited to one of the sexes alone. Again, this finding contradicts the study by Kirwin and Anderson [4], which revealed that women were less likely to migrate than men.

**Table 10:** Chi‐square analysis of sociodemographic factors and the willingness of nurses to be exported.

Willingness of nurses to be exported by the government		
Variable	Chi‐square	*p* value
Sex	2.642	0.104
Age	6.195	0.045
Religion	5.356	0.069
Marital status	1.761	0.185
Children	4.340	0.037
Educational level	0.243	0.622
Professional background	0.194	0.979
Income	0.026	0.871

The analysis of age and willingness to be exported yielded a chi‐square value of 6.195 and a *p* value of 0.045, indicating a statistically significant relationship between age and willingness to migrate among nurses in Ghana. This suggests that age influences nurses’ decisions to seek international employment, with younger nurses likely exhibiting a higher willingness to migrate compared to their older counterparts. Younger nurses, often at the early stages of their careers, may be more open to migration due to fewer family responsibilities, greater adaptability, and aspirations for career advancement. Conversely, older nurses, who may have established families, job stability, or community ties, might be less inclined to relocate. This finding highlights the importance of age as a key demographic factor in understanding migration trends among nurses in Ghana.

In this study, the analysis of religion and willingness to be exported produced a chi‐square value of 5.356 and a *p* value of 0.069, indicating that there is no statistically significant relationship between religion and willingness to migrate among nurses in Ghana. This suggests that while religious beliefs and practices may influence individual decisions, they do not play a decisive role in determining whether a nurse chooses to seek employment abroad. Factors such as economic opportunities, career growth, and personal aspirations likely have a greater impact on migration decisions than religious affiliation. However, religion may still shape destination preferences, as some nurses may prefer countries where they can comfortably practice their faith. Overall, the findings indicate that religion does not significantly affect nurses’ willingness to be exported, emphasizing the dominance of other socioeconomic factors in migration decisions.

Also, the analysis of marital status and willingness to be exported yielded a chi‐square value of 1.761 and a *p* value of 0.185, indicating that there is no statistically significant relationship between marital status and the willingness to migrate among nurses in Ghana. This suggests that whether a nurse is single or married does not significantly determine their decision to seek international employment. While it is often assumed that single nurses may be more willing to migrate due to fewer family obligations, and married nurses may face constraints related to spousal and childcare responsibilities, the findings indicate that these factors do not have a strong statistical impact on migration decisions.

The analysis of dependents (children) and willingness to be exported yielded a chi‐square value of 4.340 and a *p* value of 0.037, indicating a statistically significant relationship between having dependents and the willingness to migrate among nurses in Ghana. This suggests that nurses with children or dependents are less likely to seek international employment, as they may face challenges related to family responsibilities, childcare, and the need for stability. On the other hand, nurses without dependents are more flexible and mobile, making them more willing to migrate for better career opportunities and financial benefits. This finding highlights that family obligations can serve as a barrier to nurse migration, influencing decisions on whether to pursue opportunities abroad.

The analysis of education, professional background, and income in relation to willingness to be exported yielded chi‐square values of 0.243 (*p* = 0.622), 0.194 (*p* = 0.979), and 0.026 (*p* = 0.871), respectively, indicating that none of these factors had a statistically significant relationship with nurses’ willingness to migrate in Ghana. This suggests that educational qualification, professional specialization, and income levels do not play a decisive role in determining migration decisions among nurses. While higher education and specialized training are often assumed to enhance mobility, the findings imply that nurses at different educational and professional levels have similar inclinations toward migration. Likewise, income levels, which might be expected to influence migration decisions, do not appear to be a determining factor, suggesting that other socioeconomic and personal motivations, such as career aspirations and working conditions, may be more influential in shaping nurses’ willingness to seek opportunities abroad.

### 3.9. The Multinomial Logistic Regression Analysis

In this study, researchers hypothesized that there is a significant statistical association between the age of nursing students and their willingness to be exported. To test this hypothesis, researchers run the multinomial logistic regression analysis.

The multinomial logistic regression analysis was conducted to determine which demographic variables significantly predict a nurse’s willingness to be exported. As shown in Table 11, the number of children emerged as a significant predictor, with a *p* value of 0.033 and an odds ratio of 2.8318. This indicates that nurses with fewer or no children are approximately 2.83 times more likely to accept being exported compared to those with more children. In other words, having fewer or no dependents is positively associated with migration willingness. This finding aligns with qualitative insights, which revealed that many nurses prefer to travel alone initially, as relocating with children or family can be financially and logistically challenging. Interviews further highlighted that nurses often choose to establish themselves abroad before bringing their families, reinforcing the impact of family responsibilities on migration decisions.

**Table 11:** Logistic regression.

Outcome	Predictor variable	Relative risk ratio (RRR)	Standard error	*z*‐value	*p* value	95% confidence interval
Agree		Base outcome				

Disagree	Children	2.8318	1.3861	2.13	0.033	1.0850–7.3909
	Age	0.8411	0.2932	−0.50	0.620	0.4247–1.6658
	Education	0.8302	0.2711	−0.57	0.569	0.4377–1.5745
	Profession	0.9031	0.1178	−0.78	0.435	0.6993–1.1662
	Sex	1.5130	0.4234	1.48	0.139	0.8742–2.6184
	_cons	0.0649	0.0412	−4.31	0.001	0.0187–0.2251

*Note: Source:* Field data, 2023.

## 4. Discussion

The findings of this study reveal several important sociodemographic determinants that influence Ghanaian nurses’ willingness to migrate for overseas employment. A key finding is the significant role of age in migration decisions, with younger nurses (18–25 years) demonstrating substantially higher willingness to migrate compared to their older counterparts. This aligns with global migration patterns, where younger professionals are more mobile due to fewer family obligations and greater career aspirations. The qualitative data support this, with younger nurses expressing eagerness for international opportunities, while older nurses cited family responsibilities as barriers. This finding corroborates previous research by Bugri [14] and the World Bank [19] on youth migration trends in developing countries.

Another critical finding concerns family dependents, which emerged as the strongest predictor of migration willingness through logistic regression analysis. Nurses without children were 2.83 times more likely to accept overseas employment, highlighting how familial responsibilities constrain mobility. This substantiates migration theories that view family obligations as significant anchors that reduce migration propensity. Interestingly, while gender did not show statistical significance in the quantitative analysis, qualitative insights revealed nuanced gender dynamics. Although nursing remains female‐dominated in Ghana, both genders expressed similar willingness to migrate, contradicting studies such as Kirwin and Anderson’s [4] that found gender disparities in migration patterns. This suggests that economic motivations may override traditional gender roles in migration decisions among Ghanaian nurses.

The study found no significant relationship between migration willingness and factors such as religion, marital status, education level, professional specialization, or income. This challenges some assumptions in migration literature about the role of these variables. For instance, while higher education is often associated with greater mobility [20], the findings of this study suggest that both diploma and degree holders in nursing share similar migration motivations. The qualitative data showed that “even though education matters in migration decisions, for the nursing profession, because it is one of the skills, the nurses do not consider their level of education as a motivating factor for them to accept to be exported” (a 26‐year‐old nursing student from the UCC). Similarly, the lack of income significance contradicts conventional economic migration theories, implying that nonmonetary factors such as career growth and international exposure may be stronger drivers for Ghanaian nurses, as was revealed in the interview with some of the participants. Although studies such as [21–23] observed in their respective works the role of religion in migration decisions, their study did not consider possible migrants who do not intend to stay in the destination countries. Also, unlike their studies, which were not about health professionals being sent on government contracts, religion would not be significant, since the nurses who will be sent on government contracts will be expatriates and would not necessarily consider the religious atmosphere of the destination country before accepting to be expected.

These findings have important policy implications. The strong willingness among younger, childless nurses suggests that targeted recruitment programs could effectively address nursing shortages in destination countries. However, the barriers faced by nurses with dependents indicate a need for family‐friendly migration policies, including relocation support and family reunification programs. The study contributes to understanding nurse migration dynamics in developing countries, particularly how sociodemographic factors interact with economic and professional aspirations in shaping migration decisions. Future research could explore how these findings compare across different healthcare professions and cultural contexts.

## 5. Conclusion and Recommendation

The study demonstrates the readiness of nurses for overseas employment, indicating that the export of nurses has become a crucial avenue for improving international relations through labour diplomacy, and improving the health conditions of citizens in recipient nations, therefore younger nurses with much energy should be priotised in the selection of the nurses. Also, to optimize nurse exportation while safeguarding Ghana’s healthcare system, policymakers should prioritize structured labour export programs for younger nurses (18–25 years) and implement family‐friendly relocation policies, including spousal visas and childcare support to facilitate mobility for nurses with dependents. Simultaneously, Ghana must strengthen domestic retention through competitive salaries, career advancement opportunities, and ethical bilateral agreements with destination countries to ensure sustainable workforce management. Regular monitoring of migration trends and public–private partnerships for rotational employment models should be established to balance individual aspirations with national healthcare needs.

## Ethics Statement

This research received an ethical letter from the University of Cape Coast’s IRB before the research was conducted.

University of Cape Coast Ethical Review Board.

Ethical Clearance ID: (UCCIRB/CHLS/2023/46).

Date Issued: November 6th 2023.

## Consent

Written and signed informed consent was obtained from all the research participants and respondents.

## Conflicts of Interest

The authors declare no conflicts of interest.

## Author Contributions

Emmanuel Nyameke, who is the first author, conceived and conducted the research for his thesis. John Ansah was the supervisor for the thesis. As the supervisor, he contributed to the methodology that was employed in this study. Isaac Defiin worked on the introduction and reworked the citations and references.

## Funding

This study did not benefit from any funding from any group or any organization.

## Data Availability

The data that support the findings of this study are available on request from the corresponding author.

## References

[1] Lenormand M. , Miguel M. S. , Ramasco J. J. , Louail T. et al., Influence of Sociodemographic Characteristics on Human Mobility, Scientific Reports. (2015) 5, no. 1, 10.1038/srep10075, 2-s2.0-84930227147. PMC4438721 25993055

[2] Reynolds J. , Wisaijohn T. , Pudpong N. et al., A Literature Review: The Role of the Private Sector in the Production of Nurses in India, Kenya, South Africa and Thailand, Human Resources for Health. (2013) 11, no. 1, 10.1186/1478-4491-11-14, 2-s2.0-84877024146. PMC3637479 23587128

[3] Kuhnt J. , Literature Review: Drivers of Migration, Why Do People Leave Their Homes? is There an Easy Answer? A Structured Overview of Migratory Determinants (No. 9/2019). (2019) .

[4] Kirwin M. and Anderson J. , Identifying the Factors Driving West African Migration, 2018.

[5] Creswell J. W. and Clark V. L. P. , Designing and Conducting Mixed Methods Research, 2018, 3rd edition, Sage Publications.

[6] Gunasekare D. U. , Mixed Research Method as the Third Research Paradigm: A Literature Review, SSRN. (2016) .

[7] Creswell J. W. and Tashakkori A. , Editorial: Developing Publishable Mixed Methods Manuscripts, Journal of Mixed Methods Research. (2007) 1, no. 2, 107–111, 10.1177/1558689806298644, 2-s2.0-84990323826.

[8] Khatun K. , Maguire-Rajpaul V. A. , Asante E. A. , and McDermott C. L. , From Agroforestry to Agroindustry: Smallholder Access to Benefits From Oil Palm in Ghana and the Implications for Sustainability Certification, Frontiers in Sustainable Food Systems. (2020) 4, 10.3389/fsufs.2020.00029.

[9] Ansah J. W. , Capital Mobility and Knowledge Diffusion From China Into Ghana’s Agricultural Sector Doctoral Dissertation, 2020, University of Cape Coast.

[10] Onwuegbuzie A. J. , Johnson R. B. , and Collins K. M. , Call for Mixed Analysis: A Philosophical Framework for Combining Qualitative and Quantitative Approaches, International Journal of Multiple Research Approaches. (2009) 3, no. 2, 114–139, 10.5172/mra.3.2.114, 2-s2.0-77958188920.

[11] Cameron R. , A Sequential Mixed Model Research Design: Design, Analytical and Display Issues, International Journal of Multiple Research Approaches. (2009) 3, no. 2, 140–152, 10.5172/mra.3.2.140, 2-s2.0-78649634677.

[12] Creswell J. W. and Clark P. , Designing and Conducting Mixed Methods Research, 2007, Sage Publications.

[13] Castillo R. , Who Is Slovin and Where and How Did the Slovin’s Formula for Determining the Sample Size for a Survey Research Originated?, 2017.

[14] Bugri M. , There Are Backlog of Trained Nurses Awaiting Posting-Coalition of Unemployed Nurses, 2022, https://www.myjoyonline.com/there-are-backlog-of-trained-nurses-awaiting-posting-coalition-of-unemployed-nurses/.

[15] Coulibaly B. S. and Page J. , Addressing Africa Youth Unemployment Through Industries Without Smokestacks: A Synthesis on Prospects, Constraints and Policies, 2021.

[16] Oishi N. , Gender and Migration: An Integrative Approach, 2002.

[17] Kanaiaupuni S. M. , Reframing the Migration Question: An Analysis of Men, Women, and Gender in Mexico, Social Forces. (2000) 78, no. 4, 1311–1347, 10.2307/3006176.

[18] Gubhaju B. and De Jong G. F. , Individual Versus Household Migration Decision Rules: Gender and Marital Status Differences in Intentions to Migrate in South Africa, International Migration. (2009) 47, no. 1, 31–61, 10.1111/j.1468-2435.2008.00496.x, 2-s2.0-59749104914. 20161187 PMC2727691

[19] World Bank , Unemployment, Youth Total (% of Total Labor Force Ages 15-24)(modeledILO Estimate), 2021.

[20] Dustmann C. and Glitz A. , Migration and Education, In Handbook of the Economics of Education. (2011) 4, 327–439.

[21] Nordin M. and Otterbeck J. , Introducing the Field: ‘Migration and Religion, Migration and Religion: IMISCOE Short Reader, 2023, Springer International Publishing, Cham, 3–17.

[22] Beckford J. A. , Religions and Migrations-Old and New, Quaderni di Sociologia. (2019) no. 80, 15–32, 10.4000/qds.2599.

[23] Ahsan Ullah A. K. M. , Huque A. S. , and Kathy A. A. , Religion in the Age of Migration, Politics, Religion & Ideology. (2022) 23, no. 1, 62–76, 10.1080/21567689.2022.2057476.

